# A contemporary genomic snapshot of Salmonella Paratyphi A in Pakistan

**DOI:** 10.1099/mgen.0.001561

**Published:** 2025-11-11

**Authors:** Elli Mylona, Junaid Iqbal, Jacqueline A. Keane, Joana Pereira-Dias, Megan Carey, Mehreen Adnan, Aneeta Hotwani, Irim Iftikhar, Seema Irfan, Stephen Baker, Farah N. Qamar

**Affiliations:** 1 Cambridge Institute of Therapeutic Immunology & Infectious Disease (CITIID), University of Cambridge, Cambridge, UK; 2 Department of Medicine, University of Cambridge School of Clinical Medicine, Cambridge Biomedical Campus, Cambridge, UK; 3 Department of Paediatrics and Child Health, Aga Khan University, Karachi, Pakistan; 4 Department of Infection Biology, London School of Hygiene & Tropical Medicine, London, UK; 5 Department of Microbiology, Chughtai Lab, Lahore, Pakistan; 6 Department of Pathology & Laboratory Medicine, Aga Khan University, Karachi, Pakistan; 7 A*STAR Infectious Diseases Labs (A*STAR IDL), Agency for Science, Technology and Research (A*STAR), Singapore 138648, Singapore

**Keywords:** enteric fever, Pakistan, phylogenetics, *Salmonella *Paratyphi A

## Abstract

*Salmonella enterica* serovar Paratyphi A is a significant but under-characterised cause of enteric fever in South Asia. In Pakistan, where the typhoid conjugate vaccine has been introduced to combat *S*. Typhi, *S*. Paratyphi A remains a prominent cause of bacteraemia, raising concerns about shifts in disease burden and antimicrobial resistance (AMR). Here, we provide a comprehensive genomic and phylogenetic analysis of 354 *S*. Paratyphi A isolates collected from three provinces in Pakistan between 2017 and early 2022. Whole-genome sequencing revealed the dominance of genotypes 2.3.3 and 2.4.5, indicating a largely stable population structure over time, and the presence of widespread fluoroquinolone-associated *gyrA* mutations. Although multidrug resistance was not detected, we identified one isolate harbouring an *acrB*-R717Q mutation associated with azithromycin resistance. Plasmid and replicon analysis revealed low prevalence of extrachromosomal elements, including cryptic plasmids with unknown function. Phylogenetic placement of these isolates in a global context demonstrated close relatedness to contemporary South Asian organisms. Our findings establish a genomic baseline for *S*. Paratyphi A in Pakistan, essential for future surveillance, AMR monitoring, and evaluating the potential impact of forthcoming paratyphoid vaccines.

**Impact Statement**

Enteric fever remains a major global health issue. *Salmonella* Paratyphi A isolation, the second most prevalent cause of enteric fever after *Salmonella* Typhi globally, has been increasing in many endemic areas over the last years. In Pakistan, where the typhoid conjugate vaccine has been introduced to combat *S*. Typhi, *S*. Paratyphi A remains a prominent cause of bacteraemia, raising concerns about shifts in disease burden and antimicrobial resistance (AMR). Aiming to contribute genomic data on this organism, we analysed the whole-genome sequences of contemporary *S*. Paratyphi A organisms and constructed a phylogeny of this population. Our data highlight the importance of investigating the molecular epidemiology of *S*. Paratyphi A and using a robust typing scheme to monitor its population structure. In anticipation of the development and introduction of vaccines targeting *S*. Paratyphi A, we have established a genomic baseline for *S*. Paratyphi A in Pakistan, essential for future surveillance, AMR monitoring and evaluating the potential impact of forthcoming paratyphoid vaccines.

## Data Summary

All short reads used in this study are available at the European Nucleotide Archive under study accession PRJEB89097. All supporting data, code and protocols have been provided within the article or through supplementary data files, as well as via references to appropriate sources.

## Introduction

Enteric fever is caused by *Salmonella enterica* subsp. *enterica* serovars Typhi and Paratyphi A, B and C and is transmitted via the faecal–oral route via contaminated food or water [1]. Countries with poor sanitation and hygiene and limited access to clean water still report enteric fever cases, commonly occurring in paediatric populations, with an estimated 11–27 million cases annually and >120,000 associated deaths globally [2].

Enteric fever is endemic in low-resource countries in South Asia, such as India and Pakistan, with an incidence rate of >200 per 100,000 people [2]. In fact, enteric fever is the most reported cause of bacteraemia in children in Pakistan, reaching 1,000 cases per 100,000 child-years in Karachi [3]. *S*. Typhi is the most common cause of enteric fever in Pakistan, with multidrug-resistant (MDR) (resistance to ampicillin, cotrimoxazole and chloramphenicol) and extensively drug-resistant (XDR) (MDR plus resistance to fluoroquinolones and third-generation cephalosporins) typhoid being particularly prevalent [4,8]. While Pakistan has seen a surge in *S*. Typhi incidence, particularly following the emergence of XDR typhoid [9], *S*. Paratyphi A remains a constant burden in Pakistan and a cause of enteric fever cases. *S*. Paratyphi A was responsible for 39.9% (>1,700 cases) of enteric fever cases in Pakistan between 2009 and 2011 [5], and phase I of the Surveillance for Enteric Fever in Asia Project (SEAP) reported 31% (>800 cases) of enteric fever associated with *S*. Paratyphi A in 2012–2014 [6,10]. Phase II of the same study reported the incidence of *S*. Paratyphi A to be 6.1% (>100 cases) of enteric fever cases in Pakistan in 2016–2019 [4], which remained constant in later years (2017–2020, 529 cases) [11].

It is estimated that ~20% of the population in Pakistan has access to safe drinking water due to the lack of good quality water sources that are often contaminated with sewage [12]. Limited access to clean water, in turn, leads to elevated cases of water-borne infections and an over-reliance on antimicrobials for non-localized febrile disease. Therefore, antimicrobial resistance (AMR) is a significant public health burden in areas with poor sanitation and food hygiene [13]. Reporting of case incidents and AMR monitoring and early pathogen detection are crucial to develop strategies for managing enteric fever, with Pakistan having been a notable example of this due to the emergence and prevalence of XDR typhoid [9,14].

Pakistan was the first country to introduce the typhoid conjugate vaccine (TCV) in the immunisation programme in 2020 in response to the emergence and alarming increase in XDR typhoid cases [15,16]. While containing *S*. Typhi post-TCV introduction has been promising [15,17], Pakistan is left at a vulnerable position with regard to protection from *S*. Paratyphi A and the possibility of *S*. Typhi being replaced by *S*. Paratyphi A [18,19]. While reports of MDR *S*. Paratyphi A are rare [20,22], resistance to key antimicrobials used for treatment is being reported. Examples include reduced susceptibility to nalidixic acid and ciprofloxacin, due to mutations in the quinolone resistance-determining region [23,27], as well as sporadic cases of ceftriaxone or azithromycin resistance in the Indian subcontinent [28,30].

Here, we complemented epidemiological and demographic findings from SEAP [31] and impact assessment of typhoid conjugate vaccine following its introduction in the routine immunisation programme of Pakistan (ITRIPP) (currently unpublished) studies with a characterization of the *S*. Paratyphi A population structure in Pakistan between 2017 and early 2022. While *S*. Typhi and *S*. Paratyphi A cause clinically indistinguishable syndromes [32], the lack of protection from TCV, the different AMR profile, and the different disease transmission and age demographics may lead to changes in disease dynamics and the choice of empirical treatment, highlighting the need for *S*. Paratyphi A monitoring in a post-XDR typhoid emergence and TCV implementation era [33]. In anticipation of novel *S*. Paratyphi A and/or *S*. Typhi/*S*. Paratyphi A bivalent vaccines that are currently in development [34], having a snapshot of the *S*. Paratyphi A population prior to introducing any paratyphoid fever management strategies in endemic locations will prove essential for monitoring their impact on the bacterial population.

## Methods

### Study sites

The *S*. Paratyphi A isolates were collected from various medical departments, encompassing inpatient, outpatient, surgical units and laboratory networks within Aga Khan University Hospital (AKUH), as well as the Kharadar General Hospital (KGH) located in Sindh and the Chughtai Laboratory Network (CLN) based in Punjab in the ongoing prospective surveillance studies, notably the SEAP and the ITRIPP at Aga Khan University. In both the SEAP and ITRIPP studies, demographic and antimicrobial susceptibility testing (AST) data were systematically collected from enrolled participants. These datasets were integrated in this study with genomic data, enabling a multi-dimensional analysis to explore correlations between epidemiological patterns and the genetic profiles of the isolates.

### Bacterial cultures/isolates

*S*. Paratyphi A isolates were initially isolated from the patient’s blood via routine microbiological methods and archived at −80 °C following rigorous biochemical and serological testing as part of SEAP and ITRIPP. AST was performed using disc diffusion (Kirby–Bauer) assay as per Clinical and Laboratory Standards Insitute (CLSI) guidelines [29] at the Infectious Disease Research Laboratory at Aga Khan University. Antimicrobials tested were ampicillin (10 µg), ceftriaxone (30 µg), chloramphenicol (30 µg), ciprofloxacin (5 µg), cotrimoxazole (1.25/23.75 µg) and cefixime (5 µg). Strains were revived on Mueller–Hinton agar plates and subjected to genomic DNA extraction.

### Genome sequencing

Genomic DNA was extracted using the DNeasy Blood and Tissue Kit, and 10 ng µl^−1^ of DNA was processed with the Illumina DNA Prep Kit with unique dual (UD) indexes and subjected to paired-end sequencing on the Illumina MiSeq platform using V3 600 cartridge to generate 300 bp paired-end reads following the manufacturer’s recommendations. Sequencing was performed either in-house using the above parameters or at Eurofins (Germany) using Illumina NovaSeq 6000S to generate 150 bp paired-end reads. Thirteen genomes were excluded from analyses due to low quality scores based on abnormal genome size (<4 or >6 million bp), assembled into >1,000 contigs, positive status for contamination using confindr [35] and/or low score in bactinspector (available at https://gitlab.com/antunderwood/bactinspector), resulting in a final total number of 354 isolates that were taken forward for analysis (Table S1, available in the online Supplementary Material).

### Phylogenetic analysis

Raw sequencing reads were mapped to *S*. Paratyphi A AKU12601 (accession: FM200053) reference genome using the nf-core bactmap pipeline v1.0.0 (https://nf-co.re/bactmap/1.0.0 [36]), adding parameters -Q -L -A to the fastp tool, to make pseudogenomes based on high-quality positions in VCF files. This reference genome was selected based on its original place of isolation in Asia, its extensive genome annotation, as well as due to its genotypic identity, belonging to genotype 2.3 (using Paratype, see below), rendering it a good benchmark for the majority of the isolates in our study. In all analyses, an *S*. Typhi isolate originating in Pakistan during the same time frame was used as an outgroup. Using the pseudogenome multi-alignment file obtained from bactmap, repetitive regions, prophages and recombinant regions were removed using remove-blocks (https://github.com/sanger-pathogens/remove_blocks_from_aln) and a pre-defined coordinates list specific for Paratyphi A (PARAREPEAT, https://github.com/katholt/typhoid), followed by a screen and removal of recombinant regions with Gubbins v3.2.0 [37]. The multi-sequence alignment file obtained from Gubbins was fed into RAxML-ng v1.1.0 [38] to infer a maximum likelihood phylogenetic tree using a GTRG+gamma model and 1,000 bootstrap pseudo-analyses. For the global collection analysis, 828 publicly available *S*. Paratyphi A sequences were used that have previously been subjected to standard quality checks [39] (Table S2), combined with 69 quality-checked newly published genomes [29,40, 41]. Repetitive regions, prophages and recombinant regions were excluded as above. The filtered Pakistan collection and global collection alignment files were combined and used to run Gubbins. The maximum likelihood phylogeny tree was inferred as above with 100 bootstraps. Outgroup rooting was performed in FigTree v1.4.4 (https://github.com/rambaut/figtree), and trees were visualized and annotated using iTOL [42]. SNP distance matrices were produced using snp-dists v0.8.2 (https://github.com/tseemann/snp-dists) using the Gubbins output files.

### Genotyping, AMR gene and plasmid and replicon identification

The Paratype genotyping tool [43] was used to assign genotypes based on raw reads. Reads trimmed for quality (--cut_mean_quality 20) and adapter sequence using fastp v0.20.1 [44] were screened for known plasmid replicons against the PlasmidFinder [45] database using ARIBA v2.14.6. The trimmed reads were assembled using SPAdes v3.15.4 [46] with k-mer values 21, 33, 43, 55, 77, 99 and the --careful option. The presence/absence of known AMR genes, as well as *Salmonella*-specific point mutations in AMR genes, was detected using AMRFinderPlus v4.0 (database downloaded 2025-03-25) [47]. For the global collection analysis, detection of known point mutations in *gyrA* and *acrB* was identified along with the genotypes using Paratype as above.

Assembly graphs (fastg files) were visualized in Bandage v0.8.1 [48]. Assemblies were manually inspected for extrachromosomal replicons and those over 1,000 bp were recorded. One of two circular replicons was identified in isolate sequences as shown; a 4,692 bp replicon was detected in seven assemblies, while a 3,692 bp replicon was found in 135 assemblies. Representative sequences of the two circular replicons identified were selected and extracted from the fastg files as fasta files: all seven sequences were investigated for the 4,692 bp replicon and a random selection of ten sequences for the 3,692 bp (which were selected for representation across the phylogenetic tree clades). These were annotated using Prokka v1.14.6 [49]. Replicon sequences from randomly selected isolates (two for each replicon) were used to create blast nucleotide databases to which the other sequences were compared. Once similarity was confirmed, these two sequences were investigated using blastn available from the National Center for Biotechnology Information (NCBI) for the presence/similarity against the reference genome (taxid: 554290) and against *Salmonella enterica* (taxid: 28901). For identification of these in the global collection, a representative of each was added to the PlasmidFinder database and the genome collection was screened as above using ARIBA on fastq files.

### Data visualization and statistical analysis

Data were formatted with dplyr v1.1.2 [50], graphs were produced using ggplot v3.4.2 [51], and chi-squared tests with a cutoff for significance of *P*<0.05 were performed in RStudio v2023.06.0+421 (Posit software, PBC).

## Results

### Epidemiological and clinical observations for *S*. Paratyphi A culture-confirmed patients

*S*. Paratyphi A organisms included in the analysis were isolated from patients presenting with acute enteric fever symptoms (including fever, vomiting, abdominal pain and headache) [4] in AKUH, KGH and CLN, between 2017 and the first quarter of 2022. Approximately two-thirds of patients were male (63.6%; 225/354), and a third were female (35.9%, 127/354). The median age of patients was 14.5 years (interquartile range (IQR) 7–24 years), with most patients being between 6 and 20 years (see also Fig. S2); demographic information was missing for two individuals.

**Fig. 1.**
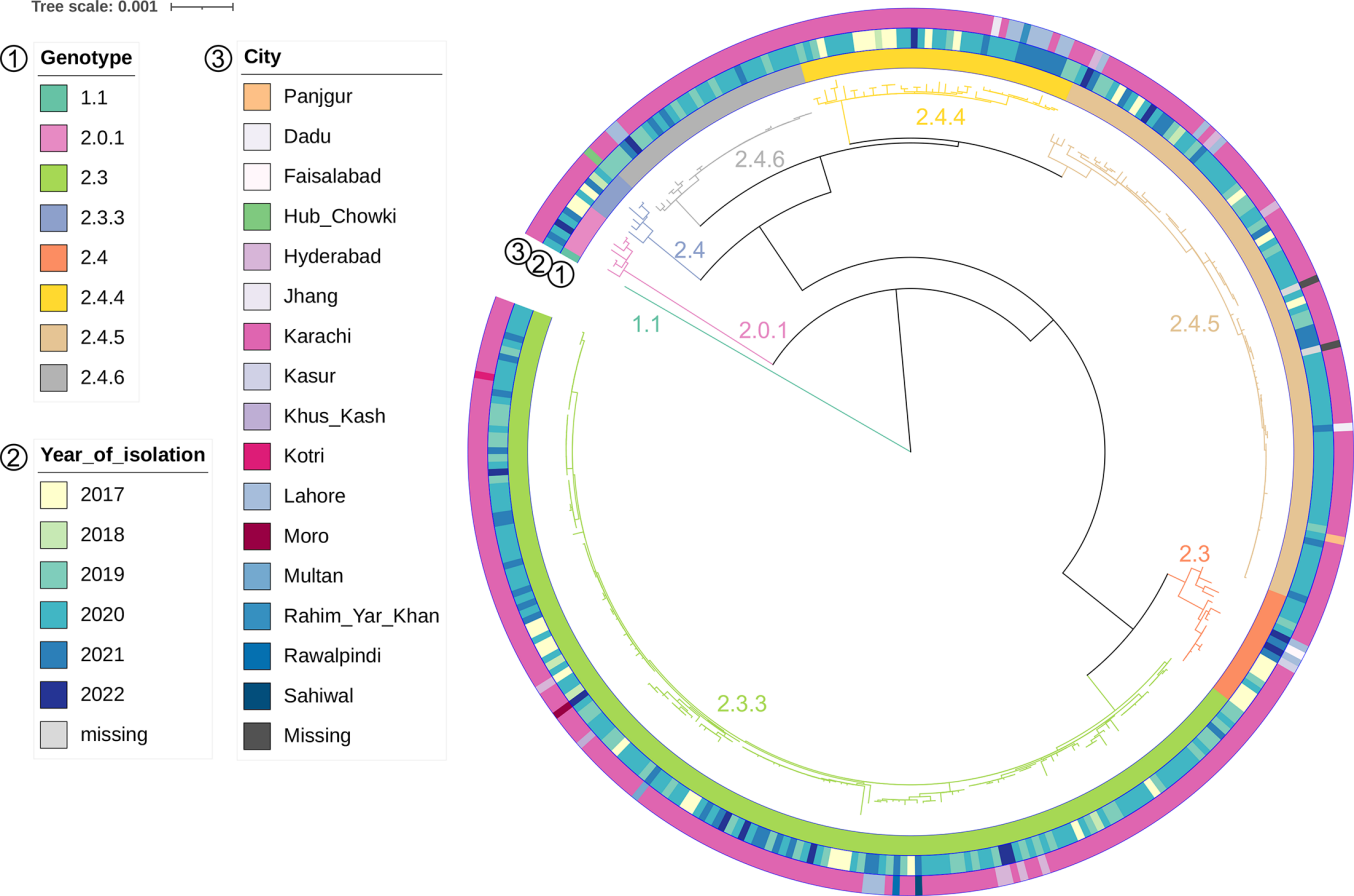
The *S*. Paratyphi A population in Pakistan in 2017–2022. Rooted SNP-based maximum likelihood phylogenetic tree of 354 *S*. Paratyphi A isolates from Pakistan, 2017–2022. The tree was constructed with the reference genome (AKU12601) included, which was removed at the visualization stage. Rings depict from inside outwards: genotype (1), year of isolation (2) and city of isolation (3) as per the legend. The branches have been coloured and annotated based on the genotype to aid in visualization.

The number of *S*. Paratyphi A organisms isolated differed for each year of our study period, with, for example, only six culture-confirmed cases being reported in 2018 (Fig. S1A). On average, *S*. Paratyphi A isolation peaked between March and May for most years, although there was a sustained number of *S*. Paratyphi A cases throughout the year (Fig. S1B). Most organisms (325/354) were isolated from within the Sindh province, most commonly from Karachi (314/354), as well as Hyderabad (8/354), Dadu (1/354), Kotri (1/354) and Moro (1/354). Cases were also reported in Punjab (24/354), specifically from Lahore (17/354), Faisalabad (1/354), Jhang (1/354), Kasur (1/354), Multan (1/354), Rahim Yar Khan (1/354), Rawalpindi (1/354) and Sahiwal (1/354). There were also three cases from the Balochistan province (one each in Hub Chowki, Panjgur and Khus Kash); geographical data were lacking for two cases (see also Fig. 1).

### *S*. Paratyphi A population structure in Pakistan

Using the genomic sequences of the *S*. Paratyphi A organisms in this collection, we inferred a SNP-based maximum likelihood phylogeny (Fig. 1) based on 1,406 SNPs and organisms were assigned genotypes using the Paratype genotyping scheme [43]. Except for one isolate belonging to genotype 1.1 and being more distantly related to the remaining organisms (median SNP distance from all other *S*. Paratyphi A isolates of 444, IQR 441–448), the collection split largely into two main subclades of primary clade 2. One subclade comprised isolates of genotyping clade 2.3 (181/354; median pairwise difference of 10, IQR 9–13): genotypes 2.3 (17/354, 4.8%) and 2.3.3 (164/354, 46.3%). The other clade consisted of isolates belonging to genotyping clade 2.4 (165/354; median pairwise difference of 19, IQR 17–57) and, specifically, 2.4 (6/354, 1.7%), 2.4.4 (40/354, 11.3%), 2.4.5 (88/354, 24.9%) and 2.4.6 (31/354, 8.8%). A small number of isolates were predicted to be genotype 2.0.1 (7/354, 2.0%) since a secondary genotyping clade could not be assigned and differed by 249 SNPs on average (IQR 247–250) upon pairwise comparison with the remaining isolates.

The genotype distribution remained largely constant throughout the study period (Fig. S2A). Overall, the plurality of isolates was genotype 2.3.3 for all study years (38.9 %–56.7 % per year), with a lower proportion of cases being caused by genotypes 2.3, 2.4, 2.4.4 and 2.4.5. Genotype 2.4.6 emerged in 2019 and remained constant in low numbers until the end of the study period. It is worth noting that the distribution of genotypes also did not vary by month in each year (Fig. S2B); we did not identify any correlation between month and genotype (chi-squared *P*=0.51, with NA values excluded). Furthermore, although we had a small number of isolates from many of the locations reported above, we did not observe any restriction of any genotype to a specific location, and all locations had a representation of reported genotypes. Lastly, the distribution of *S*. Paratyphi A genotypes did not vary by gender (chi-squared *P*=0.63, Fig. S2C), and all genotypes were identified in all age brackets of the population under study (chi-squared *P*=0.22, Fig. S2D).

### Antimicrobial susceptibility

**Fig. 2.**
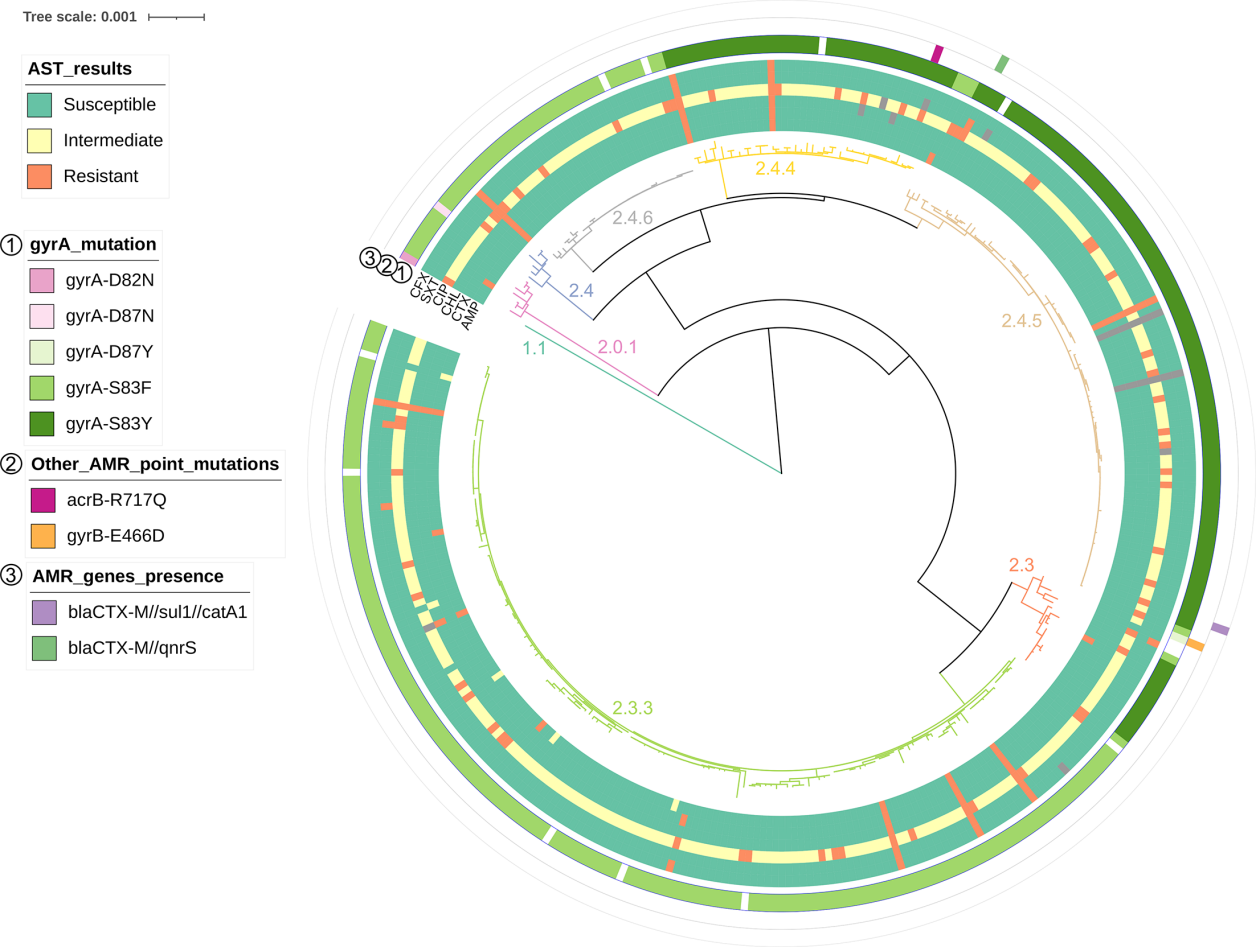
AMR susceptibility and genomic markers. AST results are shown in the inner rings, as tested for ampicillin (AMP), ceftriaxone (CTX), chloramphenicol (CHL), ciprofloxacin (CIP), cotrimoxazole (SXT) and cefixime (CFX). Grey bands indicate not available phenotypic testing. Rings 1–3 show genomic AMR markers: point mutations in *gyrA* (1), other point mutations (2) and the presence of AMR genes (3). Blank (white) bands indicate the absence of an identified mutation. Branch colour and annotation indicate genotype branches as identified in Fig. 1.

The *S*. Paratyphi A isolates were tested for their phenotypic susceptibility to various antimicrobials by disc diffusion. Most isolates were susceptible to ampicillin (97.7%; 346/354), ceftriaxone (99.4%; 352/354), chloramphenicol (98.9%; 350/354), cotrimoxazole (98.9%; 350/354) and cefixime (99.2%; 351/354) (Fig. 2). In contrast, 83.3% (295/354) of the isolates showed intermediate resistance, and 15.0%(53/354) were resistant to ciprofloxacin (Fig. 2).

We next confirmed the genomic markers for AMR in *S*. Paratyphi A (Table S1, Fig. 2). In accordance with the phenotypic results, 96.6% of isolates carried one of five SNPs in *gyrA*; of these, 95.8% (339/354) had a substitution at amino acid S83, either S83F (204/354; 57.6%) or S83Y (135/354; 38.1%), two isolates had a substitution at amino acid D87 [D87Y or D87N (0.3% each)] and one isolate carried a D82N substitution. We also identified one isolate carrying the *acrB*-R717Q mutation that confers reduced susceptibility to azithromycin; however, we did not have phenotypic results to corroborate this. Two isolates also carried additional AMR genes: one carried *blaCTX-M* (55.7% coverage with 98.8% identity), *sul1* (81.7% coverage with 100% identity) and *catA1* (50.2% coverage with 100% identity) and the other one *blaCTX-M* (50.9% coverage with 99.3% identity) and *qnrS* (53.3% coverage with 100% identity) (Fig. 2). It is worth noting that discrepancies between genotypic and phenotypic AMR profiles may be a result of limitations in AMR databases, resistance mediated by changes in gene expression levels and/or unknown resistance mechanisms. To exclude variation in phenotypic testing, we repeated some of the AST tests, but these discrepancies could not be resolved.

### Plasmids and extrachromosomal replicons

Finally, we investigated whether the *S*. Paratyphi A population in Pakistan carried any known plasmids or other replicons identified as extrachromosomal circular contigs in assemblies. We identified several isolates carrying a *col* type plasmid (ColRNAI or Col_pHAD28, Fig. S3). ColRNAI was restricted to genotype 2.0.1 *S*. Paratyphi A isolates and was carried by all such organisms in our collection. The Col_pHAD28 plasmid was detected in a small number of genotype 2.3.3 organisms.

We also investigated the genome assemblies for extrachromosomal circular contigs and found that 40.1% of genomes carried either a 4,692 bp long (7/354) or a 3,692 bp long (135/354) replicon (Fig. S3). The former was strictly associated with 2.0.1 organisms, and all such organisms in the collection carried this replicon. The smaller 3,692 bp circular replicon was carried by mostly 2.3.3 *S*. Paratyphi A organisms (128/135), but also sporadically by isolates of genotypes 2.3 (2/135), 2.4.4 (1/135), 2.4.5 (3/135) and 2.4.6 (1/135). Both replicons were absent from the reference *S*. Paratyphi A AKU_12601 genome, as assessed by sequence comparison. Annotation of representative 4,692 bp replicons revealed that they contained coding sequences for at least one hypothetical protein, regulatory protein rop, DNA relaxase MbeA and mobilization protein MbeC. The 4,692 bp replicon sequence was highly comparable (>99%) to plasmids found in other *Salmonella* serovars (blast hits), which also encoded for a similar set of proteins. Annotation of the 3,692 bp replicon indicated two to three ORFs encoding hypothetical proteins. Blasting of two such replicon sequences against *S. enterica* revealed that it was identical to the cryptic pGY1 plasmid previously found in a *S*. Paratyphi A clinical isolate from China detected in 2009 [52].

### The Pakistani *S*. Paratyphi A population in a global context

**Fig. 3.**
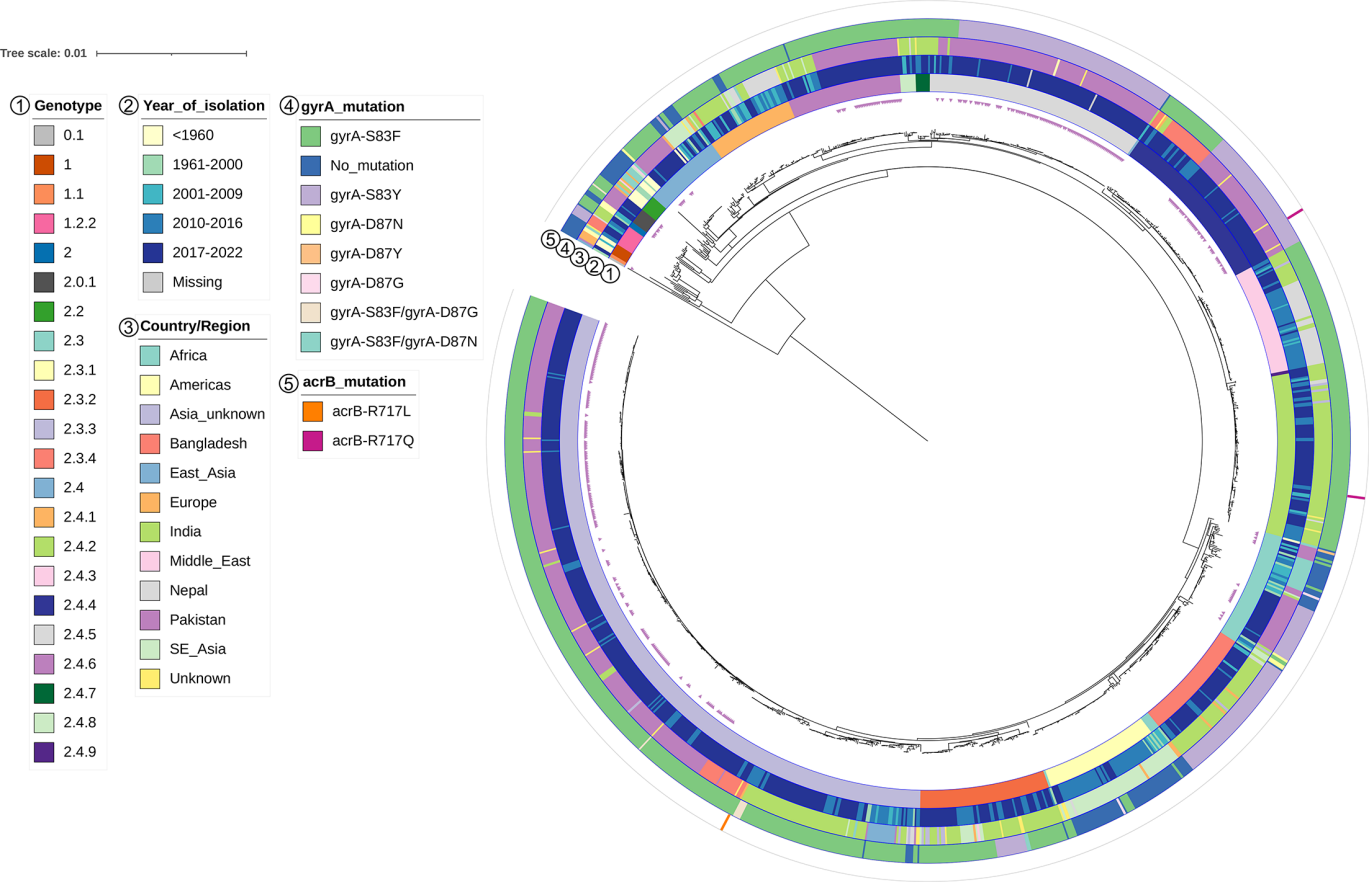
The Pakistani *S*. Paratyphi A collection in a global context. Rooted SNP-based maximum likelihood phylogeny of *S*. Paratyphi A from our collection (marked with purple arrows in the innermost ring) along with a collection of 895 global representative *S*. Paratyphi A isolates. Annotation rings show from inside: genotype, year of isolation, country/region of origin, *gyrA* mutation and *acrB* mutation as per the legend. A tree was constructed with the reference genome (AKU_12601) included, which was removed at the visualization stage. For visualization purposes, the tree was re-rooted at the midpoint of the longest branch.

We next constructed a global phylogeny based on 7,027 SNPs by combining our present collection with 895 publicly available *S*. Paratyphi A genomes from a wide range of geographical regions and chronological time frames (Table S2). As predicted, the genomes from the present collection clustered with global isolates sharing the same genotype (Fig. 3). Additionally, the collection of genomes from Pakistan was most closely related to contemporary isolates, i.e. isolates from 2017 to 2022, as well as 2010–2016, originating from Bangladesh or India, and other Asian locations, with few exceptions (such as Africa) (Fig. 3).

The global analysis also confirmed the wide presence of point mutations in *gyrA*. The one isolate we identified to carry a point mutation within *acrB* was positioned distantly from two other isolates carrying point mutations in this gene that were also from different locations (one from Bangladesh and one from India).

## Discussion

Pakistan is a major hotspot for enteric fever. XDR *S*. Typhi is a major public health concern with various interventions currently underway aiming to reduce the disease burden. Conversely, *S*. Paratyphi A has been neglected despite a considerable proportion of enteric fever cases caused by this pathovar in Pakistan [4,6,11]. A vaccine against *S*. Paratyphi A is currently lacking, but several are in development [34]. Similarly, a robust framework for sustained surveillance to support investigation of population dynamics has only recently begun to be implemented with efforts including the development of Paratype [43,53]. In anticipation of vaccine introduction, it is important to have a defined *S*. Paratyphi A population structure in countries where a vaccine will be most likely of high priority. To this end, we collated a collection of *S*. Paratyphi A organisms from various studies previously conducted across Pakistan (SEAP and ITRIPP), performed whole-genome sequencing and phylogenetic analysis and defined the population structure at the genomic level.

The *S*. Paratyphi A organisms in this collection were isolated within a 4.5-year period (2017–2022). Infected patients were mostly male with a median age of 14.5 years, including slightly older children but still within the at-risk age range identified during the first phase of the SEAP study [6]. The slightly higher proportion of enteric fever in males is commonly reported in many other countries in South Asia, like India, Nepal and Bangladesh and may be a result of differential gender behaviours related to exposure risk and healthcare seeking [9]. Furthermore, included cases indicated a consistently high isolation rate across the year, with a peak in the spring months of each year, such as an average peak during March and April, which was earlier in the year compared to other observations in the country [4,54]. This could have been a result of healthcare-seeking behaviour following the winter months and/or sampling factors, including stochastic variation in case numbers.

While most isolates were isolated within Karachi, we included isolates from other provinces and cities to obtain a better representation of the *S*. Paratyphi A population in Pakistan. Our characterization of the *S*. Paratyphi A population in Pakistan suggested that the composition of the population remained largely consistent during the study period, with comparable representation of genotypes and genomic traits across locations and time. Most isolates belonged to clades 2.3 or 2.4, and the composition of genotypes remained largely constant across the study period, suggesting a constant level of genetic diversity that is maintained by the current environmental pressures and/or by re-introduction and exchange of genotypes. In fact, the genotypes circulating in Pakistan during the study period were like those found in neighbouring countries, possibly the result of travelling and movement in the Indian subcontinent.

While a high incidence of MDR *S*. Typhi is recorded in many endemic settings in Asia and Africa [24,55, 56], MDR is generally absent in *S*. Paratyphi A with a few exceptions [21]. Here, most organisms were resistant or had reduced susceptibility to fluoroquinolones, which is associated with point mutations in *gyrA* at codons 83 and 87 [26]. Due to increased use of fluoroquinolones, mutations in *gyrA* are commonly found in *S*. Paratyphi A globally. We observed the majority of isolates carrying a mutation in S83, the most frequently detected mutated site in *S*. Paratyphi A and in particular in genotypes including 2.3.3 and 2.4.4 [43]. The population in the study here also reflected that mutations at codon 87, as well as double mutations (at codons 83 and 87), are rarer [43]. While S83F has previously been shown to be characteristic of 2.4.4, with S83Y being less common in this genotype, most isolates belonging to this genotype in this study, as well as 2.4.5, carried S83Y, suggesting that this mutation may be expanding in the country [43]. Notably, we identified one isolate carrying a point mutation in *acrB*, which is linked to resistance to azithromycin [29]. Mutations in this gene have been previously identified in *S*. Paratyphi A in Bangladesh [29,57], and azithromycin-resistant *S*. Typhi and *S*. Paratyphi A have been recorded in Pakistan [28,58]. It is unclear whether this isolate spontaneously acquired this mutation or whether it was introduced from neighbouring countries, but the former seems likely given that the isolate was not closely related to any of the global isolates carrying a mutation in *acrB*. However, *acrB* mutations in *S*. Paratyphi A 2.4.4 organisms have been recorded [43]. Therefore, monitoring the spread of such mutations and AMR profiles is crucial.

*S*. Paratyphi A does not commonly carry plasmids [43]. We found a small set of isolates carrying *col*-type plasmids, as well as one of two extrachromosomal replicons. While *col* plasmids typically encode for bacteriocins and their antidotes, ColRNAI and Col_pHAD28 have been found to carry antibiotic resistance genes in *Klebsiella* and *Escherichia coli* [59,60]. ColRNAI is non-mobilizable and relies on other conjugative plasmids to move between organisms. Specifically, ColRNAI was identified only in 2.0.1 organisms in our collection, which also carried a 4,692 bp replicon that is predicted to encode for mobilization proteins such as MbeAC. It is plausible that the one plasmid facilitated the conjugation of the other in these organisms. Alternatively, several isolates belonging to genotypes 2.3.3, 2.4.4, 2.4.5 or 2.4.6 carried a 3,692 bp extrachromosomal circular contig that was comparable to pGY1, a cryptic plasmid previously identified in *S*. Paratyphi A in China that shares partial homology to other cryptic plasmids in other *Salmonella* serovars and *Klebsiella* [52]. While the importance of these plasmids remains obscure, recording their occurrence and abundance in *S*. Paratyphi A will facilitate understanding of important aspects like plasmid stability in this pathogen and potential acquisition of AMR gene-carrying plasmids.

This study has limitations that should be considered when interpreting the findings. First, the collection of *S*. Paratyphi A isolates was dependent on patients presenting to healthcare facilities, introducing potential bias towards individuals with greater healthcare access, particularly in urban centres. Consequently, genotypes circulating in more remote or rural populations – where diagnostic capacity may be limited – could be underrepresented, limiting the geographic comprehensiveness of our analysis. Second, although our dataset includes isolates from multiple provinces, a substantial proportion originated from Karachi. This urban concentration may have skewed the observed genotype distribution and may not fully reflect the diversity of circulating organisms across Pakistan. Despite these limitations, our study provides the most extensive genomic characterization of *S*. Paratyphi A in Pakistan to date. It establishes a critical baseline for ongoing surveillance, particularly in the context of expanding vaccine coverage and rising antimicrobial resistance, and highlights the importance of broadening sampling efforts in future studies to capture a more representative picture of the national pathogen landscape.

In summary, we characterized the recent population structure of *S*. Paratyphi A in Pakistan. We recorded a relatively constant composition of genotypes and AMR profile through time. Given the overwhelming burden and likely mobilization of AMR genes in South Asia, a critical question is why *S*. Paratyphi A seems incapable of acquiring a similar compendium of resistance elements as those observed in *S*. Typhi in Pakistan, despite their co-circulation. However, we observed a single occurrence of resistance to azithromycin, emphasizing the importance of sustained monitoring of the population. Ongoing genomic surveillance is crucial for informing treatment guidelines, vaccine introduction and global health preparedness, by tracking AMR and detection of relevant mutations early, as well as monitoring antigenic and genotypic shifts.

## Supplementary material

10.1099/mgen.0.001561 Uncited Supplementary Material 1.

10.1099/mgen.0.001561 Uncited Supplementary Material 2.

## Abbreviations

AKUH: Aga Khan University Hospital
AMR: antimicrobial resistance
AST: antimicrobial susceptibility testing
CLN: Chughtai Laboratory Network
ITRIPP: impact assessment of typhoid conjugate vaccine following its introduction in the routine immunization programme of Pakistan
KGH: Kharadar General Hospital
MDR: multidrug resistance
SEAP: Surveillance for Enteric Fever in Asia Project
*S.* Paratyphi A: *Salmonella enterica* subsp. *enterica* serovar Paratyphi A
S. Typhi: *Salmonella enterica* subsp. *enterica* serovar Typhi
TCV: typhoid conjugate vaccine
XDR: extensively drug-resistant
