# Supplementary material for: A contemporary genomic snapshot of Salmonella Paratyphi A in Pakistan
Source: Microb Genom. 2025 Nov 11;11(11):001561. doi: 10.1099/mgen.0.001561 (PMC12604761; doi:10.1099/mgen.0.001561)
Supplement: Uncited Supplementary Material 1. [file mgen-11-01561-s001.pdf]

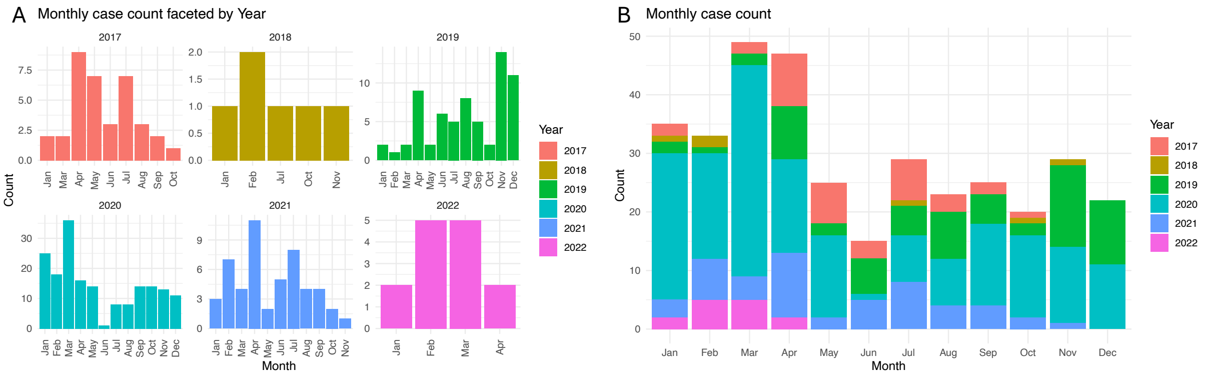

Figure S1. Annual and monthly distribution of *S.* Paratyphi A cases in Pakistan during the study period.

(A) Bar plots showing monthly cases recorded each year of the study period. (B) Stacked bar plot depicting the total monthly distribution of cases throughout the study period. Demographic information was missing for two cases (NA).

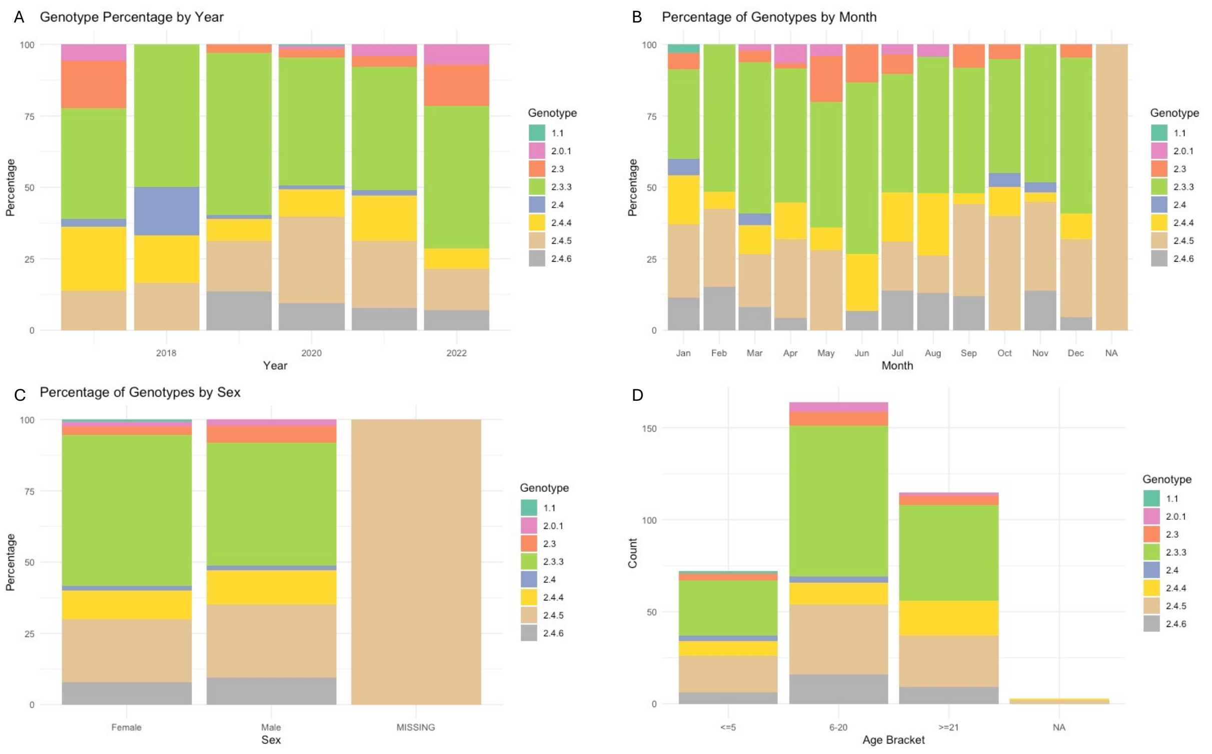

Figure S2. Distribution of *S.* Paratyphi A genotypes in Pakistan.

Stacked bar plots showing the distribution of genotypes (A) by year, (B) by month, and (C) by sex shown as percentage of total number of isolates, and (D) by age bracket shown by isolate count. Isolates for which this information (year) was missing were excluded.

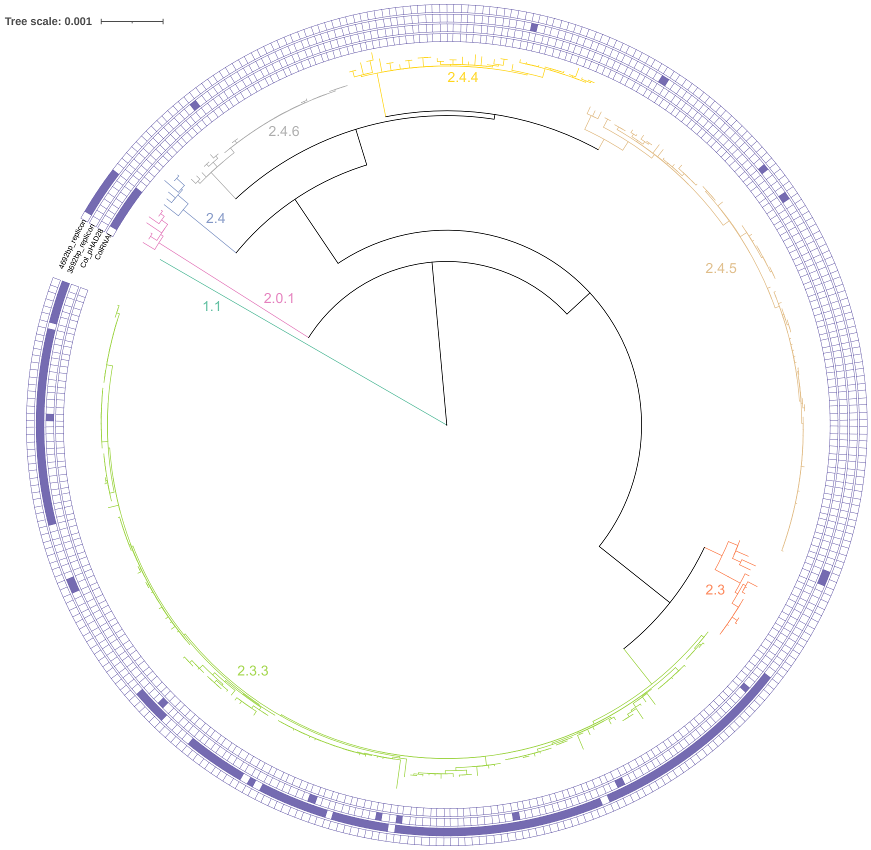

Figure S3. Plasmid and extrachromosomal replicon identification in the Pakistani *S*. Paratyphi A population.

Rings indicate presence (filled rectangle) or absence (outline) of plasmids/replicons as indicated: ColRNAI, Col_pHAD28, and the identified 3,692 bp and 4,692 bp long extrachromosomal replicons. Branch colour and annotation indicate genotype branches as identified in Figure 1.
